# Sex-specific skeletal muscle fiber atrophy and reduced macrophage inflammation in a rodent transverse aortic constriction model

**DOI:** 10.1007/s00424-026-03220-w

**Published:** 2026-10-10

**Authors:** William S. Evans, Maria Canellas da Silva, Gabriel Pena, Sarah Kuzmiak-Glancy, Steven J. Prior

**Affiliations:** 1 Department of Kinesiology, University of Maryland, College Park, MD 20742 USA; 2 Department of Exercise Science, Elon University, Elon, NC USA; 3 Department of Veterans Affairs, Baltimore Veterans Affairs Geriatric Research, Education and Clinical Center, Baltimore, MD USA

## Abstract

Heart failure is associated with systemic skeletal muscle remodeling, where sex-specific differences suggest differential peripheral involvement in disease progression. Skeletal muscle macrophages regulate angiogenesis and tissue remodeling and may contribute to early alterations in muscle structure and function during cardiac pressure overload. We tested whether chronic pressure-overload induces sex-specific skeletal muscle remodeling and alters macrophage abundance and paracrine signaling in male and female Sprague Dawley rats that underwent transverse aortic constriction (TAC) or sham surgery at 4 weeks of age and were studied after 40 wk. TAC induced 12% greater heart mass (*p* = 0.049) and 17% greater heart-to-body mass ratio (*p* = 0.018) in males, while females showed minimal cardiac remodeling. In extensor digitorum longus (EDL) muscle, TAC males exhibited ~ 10% smaller fiber perimeter corresponding with higher capillary-to-fiber perimeter exchange (*p* < 0.05 for both), while female TAC rats had no difference in fiber size and 17% lower capillary-to-fiber ratio than female sham (*p* = 0.033). In soleus muscle, TAC rats had higher capillarization indices (*p* < 0.05) regardless of sex. Muscle macrophage abundance was 19% lower in TAC males than sham males (*p* = 0.005). TAC macrophages secreted less MCP-1 (− 35%, *p* = 0.0058), IL-1β (− 16%, *p* = 0.0167), TNF-α (− 16%, *p* = 0.0337), and VEGF-A (− 16%, *p* = 0.0120) and produced less complex endothelial networks (18% larger mesh size, *p* = 0.001). These findings indicate that TAC is associated with skeletal muscle remodeling and macrophage immunosuppression, with some changes occurring in a sex-specific manner. This suggests the potential for immune-microvascular coupling in peripheral adaptations to cardiac pressure overload.

**Supplementary Information:**

The online version contains supplementary material available at https://doi.org/10.1007/s00424-026-03220-w.

## Introduction

Heart failure is increasingly recognized as a systemic syndrome in which peripheral tissues, including skeletal muscle, undergo significant remodeling alongside cardiac dysfunction. Skeletal muscle abnormalities such as microvascular rarefaction and dysfunction contribute to reduced exercise capacity in heart failure [1–3]. Our previous work using a transverse aortic constriction (TAC) model of cardiac pressure overload identified modest cardiac dysfunction but early, sex-specific alterations in skeletal muscle fiber size [4]. These findings suggest that peripheral remodeling may emerge early during disease progression, prior to overt cardiac impairment. We therefore posit that regulation of muscle fiber size by factors such as skeletal muscle capillarization is disrupted during pressure overload due to an imbalance between pro- and anti-angiogenic processes.

Capillaries serve as the primary interface for oxygen and substrate delivery and are essential for maintaining muscle mass and metabolic function [5]. In parallel, tissue-resident macrophages regulate capillarization and muscle repair by modulating the magnitude and resolution of inflammatory signaling [6–10]. As such, macrophages may act as upstream regulators of capillary density and myofiber size, two features that influence substrate delivery, ATP production, and overall muscle function during early heart failure development. Although systemic inflammation is implicated in heart failure, it remains unclear how inflammatory signaling shapes macrophage behavior, capillarization, and skeletal muscle remodeling in response to TAC, and whether these processes differ by sex.

Inflammatory cytokines such as tumor necrosis factor-alpha (TNF-α) [11, 12], interleukin 1-beta (IL-1β) [13, 14] and monocyte chemoattractant protein-1 [15] (MCP-1) regulate processes central to muscle remodeling, including macrophage recruitment. These macrophages drive processes influencing endothelial recruitment and angiogenesis, sarcomere protein turnover, fibroblast activation and inhibition, and the resolution of acute neutrophil-mediated inflammation [16]. Chronic, systemic inflammatory signals may disrupt the resolution aspect of these processes [17], and can also disrupt angiogenic signaling, eventually contributing to smaller muscle fibers and reduced force production. Overall, altered macrophage activity provides a plausible link between systemic inflammation, microvascular dysfunction and skeletal myofiber remodeling in cardiac pressure-overload. Therefore, the purpose of this study was to extend our previous findings and evaluate macrophage infiltration, phenotype, and angiogenic potential as well as skeletal muscle capillarization and fiber size to determine whether immune–microvascular coupling explains sex-specific alterations to skeletal muscle in TAC rats.

## Methods

### Animals

Male and female Hilltop Sprague-Dawley^®^ (SD) rats (Rattus norvegicus, Hilltop Lab Animals, Inc.) were used. All animal research procedures described below were approved by the University of Maryland Institutional Animal Care and Use Committee (IACUC).

### Surgical procedures

Male and female rats were subjected to a TAC or Sham surgery procedure at 4 weeks of age as previously described [18]. Briefly, all instruments were cleaned and sterilized, rats were anesthetized, administered analgesic [buprenorphine (0.05–0.1 mg/kg)], placed in the supine position, and the surgical area was shaved and disinfected. A horizontal skin incision was made at the suprasternal notch region, and a longitudinal cut was made in the proximal part of the sternum. A 4 − 0 silk suture attached to a blunted-needle was passed around the aortic arch between the origin of the right innominate and left common carotid arteries and tightened around a 20-gauge needle. After ligation, the needle was quickly removed, and the surgical area was closed. In Sham controls, the same procedures were followed, however the aorta was not constricted. Rats were euthanized after 40 weeks via exsanguination.

### Tissue collection and handling

After animals were sacrificed, the soleus, extensor digitorum longus (EDL) and quadriceps muscles were excised. The soleus and EDL were immediately massed, and tissue cross sections were embedded in Tissue-Tek^®^ O.C.T. (Sakura, Dover, DE), frozen on liquid nitrogen, and stored at -80 °C until processing and analysis. The quadriceps muscles were immediately washed with ice-cold Dulbecco’s Modified Eagle Medium (DMEM) and processed for muscle digestion and immuno-fluorescent cell sorting as described below.

### Isolation of skeletal muscle macrophages and fluorescence-activated cell sorting

To isolate macrophages, freshly excised quadriceps muscles were kept on ice and dissected free of visible fat, tendons, and fascia. Samples were minced and ~ 1.5 g of muscle tissue were digested in serum free DMEM with the Miltenyi Muscle Digestion kit (Miltenyi Biotech, Auburn, CA) according to manufacturer guidelines. Briefly, samples were rotated at 37 °C for 30 min and vortexed every 10 min to dislodge muscle pellets. During the next 30 min of digestion, samples were triturated with a wide bore pipette to aid in gentle mechanical digestion. Samples and buffers were placed on ice for the remainder of the procedure. Homogenates were passed through a 100 μm filter and washed with 10–15 mL of fresh serum-free DMEM, then spun at 500 x g for 20 min at 4 °C to remove platelets and cell debris. Red blood cells were lysed, and samples were washed with cell staining buffer (CSB) containing 2% FBS, 10mM EDTA in Ca^++^ and Mg^+^ free PBS. Homogenates were incubated with pre-titrated volumes of anti-rat FcR blocker (BD Biosciences Cat# 550271, RRID: AB_393568) in CSB followed by staining with pre-titrated amounts of anti-CD11b Pacific blue (Thermo Fisher Scientific Cat# 11-0112-85, RRID: AB_464936), anti-MHCII PerCP-eFluor 710 (Thermo Fisher Scientific Cat# 46-0463-82, RRID: AB_10736599) and anti-rat CD45 PE/Cyanine7 (BioLegend Cat# 202214, RRID: AB_2253306) and Rat CD31 BV510 (BD Biosciences Cat# 744358, RRID: AB_2742177) in CSB. Samples were washed and passed through a 30-µm filter. Sorting was performed on a FACS Aria II Cell Sorter (BD Biosciences). Appropriate single stain controls and fluorescence minus one (FMO) controls were run for compensation and gating. Doublets and debris were gated out and CD45^+^CD11b^+^MHCII^+^ macrophages and CD45^−^CD31^+^ were selected [19]. The relative and absolute counts CD45^+^CD11b^+^MHCII^+^ were compared. In examining these populations and subpopulations the relative distribution of macrophages and myeloid cells can be ascertained. Our data showed ~ 85% purity after sorting of cells. CD45^+^CD11b^+^MHCII^+^ cells were washed and resuspended in a 96-well plate at a density of 5000 cells/100µL of media. To investigate macrophage activation, TAC or Sham skeletal muscle macrophages were incubated for 48 h in DMEM + 10% FBS alone or with 1 µg/mL lipopolysaccharide (LPS) to elicit classically activated macrophages [7]. Cells were spun at 500 x g for 5 min and conditioned medium was harvested and frozen at -80 °C for future analyses.

### Muscle capillarization and fiber size

Samples were placed in a cryostat at -20 °C and mounted. Muscle orientation was determined and 10–15 μm transverse sections were cut and placed on 10 mm glass slides. Sections were fixed and permeabilized with ice-cold acetone for 7 min and washed 3 times with PBS. After washing, tissue was blocked with 5% goat serum in PBS for 1 h at room temperature followed by 3 washes in PBS. *Griffonia Simplicifolia Lectin I* Dylight 594 (Vector Laboratories Cat# DL-1207, RRID: AB_2336415) (5 µg/mL) and rabbit anti-rat Laminin (Abcam Cat# AB_11575, RRID: AB_298179) (5 µg/mL) primary antibodies were added for 30 min at room temperature. After washing with PBS, the secondary antibody anti-rabbit AF488 (Thermo Fisher Scientific Cat# A-11034, RRID: AB_2576217) was added at 1 µg/mL for 30 min at room temperature followed by 3 washes with PBS. Sections were mounted with ProLong™ Gold Antifade Mountant with DAPI (Thermo Fisher Scientific) and immediately imaged with a Zeiss Axio Observer (Zeiss International) fluorescent microscope. Three randomly-selected regions of each muscle cross-section were used for analyses, with 102 ± 18 total fibers analyzed per sample. As previously published [20–22], indices of capillarization and muscle morphology were determined, including (1) capillary contacts per fiber, (2) individual capillary-to fiber ratio, (3) capillary density (4) muscle fiber perimeter, 6) muscle fiber cross-sectional area. Sample immunostaining images of portions of EDL and soleus muscles are shown in Supplemental Fig. 2. To measure capillarization, images were processed with the Muscle2View (CellProfiler, Broad Institute of MIT and Harvard, MA) pipeline [23]. Data were sorted and manually validated by crosschecking post-processing images with manual measurements.

### Measurement of macrophage cytokine concentrations

For processing of inflammatory signals, samples were thawed on ice and a multiplex assay were performed according to manufacturer guidelines (Procartaplex Th1 8 plex, Invitrogen) for granulocyte-monocyte colony stimulating factor (GM-CSF), interferon gamma (IFN-g), interleukin (IL)-6, tumor necrosis factor alpha (TNFα), IL-1B, and monocyte chemoattractant protein-1 (MCP-1). GM-CSF, IL-6 and IFN-gamma were not detected in our sample and are not reported. Samples were analyzed on a Luminex Magpix (Luminex Corporation) multiplex plate reader and values are reported as median fluorescence intensity.

### Angiogenic tube formation assay

To perform angiogenic tube formation assays, pooled human umbilical vein endothelial cells (HUVECs, Lonza) were first expanded in endothelial growth medium supplemented with growth factors and serum (EGM-2, Lonza) treated with antibiotic-antimcotic (Invitrogen) to prevent contamination. Cultured plates were coated with Reduced-Growth Factor Matrigel (BD Biosciences) and incubated at 37 °C and 5% CO_2_ until solidified (~ 1 h). 20,000 endothelial cells were plated for each condition. We optimized the amount of macrophage conditioned media needed to induce changes in tube formation without cell death that washed out conditional effects. As a result, 20µL of macrophage conditioned medium was added to 180 µL in EGM-2 and applied to HUVECs. After 24 h, the center of the wells was visualized under a light microscope at 200X magnification and imaged (DS-Ri2 Nikon Eclipse Ti-U, Nikon). Finally, the images were processed with the Angiogenesis Analyzer for ImageJ [24]. Total segment length, number of junctions, mean mesh size, number of segments and number of meshes were determined to ascertain network complexity and structure.

### Statistical analysis

Statistical analyses were performed using PRISM (Version 9). Initially, males and females were grouped, and effects of surgery were compared with a one-way ANOVA. We also compared the effects of sex and TAC with a 2 × 2 two-way ANOVA. Skeletal muscle masses and cardiac data were only compared between TAC and Sham within sex because of inherent size differences between sexes and the non-allometric scaling of tissue mass to body size. In these scenarios, comparisons between TAC and Sham rats were performed within sexes (e.g., male Sham vs. male TAC and female Sham vs. female TAC) using independent t-tests.

## Results

### Characteristics of TAC and Sham rats

Characteristics of the male and female TAC and Sham rats were previously published [4] and select data are shown here in Table 1. In general, heart size was greater in male TAC vs. Sham rats, but these differences were not observed in female rats. Male TAC rats had 12% greater heart mass (*p* = 0.049), 17% greater heart to body mass ratios (*p* = 0.018), and a tendency for 10% greater mean left ventricular width to body mass ratios (*p* = 0.054) than in Sham rats. Neither female nor male TAC rats demonstrated lower ejection fractions than Sham rats. Contrary to the heart, there were no significant differences in skeletal muscle mass between male Sham or male TAC rats.

**Table 1:** Skeletal muscle and cardiac masses in male and female TAC and Sham rats

	Male		Female	
	Sham (*n* = 15)	TAC (*n* = 17)	Sham (*n* = 12)	TAC (*n* = 11)
Body Mass (g)	621 ± 52	598 ± 45	311 ± 22	301 ± 29
Heart Mass (g)	1.74 ± 0.23	1.95 ± 0.34 *	1.14 ± 0.22	1.18 ± 0.19
Heart to Body Mass (g/kg)	2.81 ± 0.39	3.28 ± 0.63 *	3.66 ± 0.55	3.95 ± 0.60
LV Width (mm)	3.89 ± 0.40	4.13 ± 0.51	3.35 ± 0.44	3.47 ± 0.35
LV Width: Body Mass (mm/kg)	6.29 ± 0.77	6.94 ± 1.02 ^†^	10.77 ± 1.13	11.65 ± 1.83
Ejection Fraction (%)	74.9 ± 7.6	72.2 ± 8.9	87.8 ± 5.3	89.4 ± 6.8
Soleus Mass (g)	0.264 ± 0.047	0.243 ± 0.035	0.145 ± 0.023	0.141 ± 0.014
EDL Mass (g)	0.251 ± 0.026	0.255 ± 0.019	0.136 ± 0.013	0.143 ± 0.034
Soleus: Body Mass (g/kg)	0.425 ± 0.074	0.408 ± 0.062	0.467 ± 0.063	0.471 ± 0.061
EDL: Body Mass (g/kg)	0.406 ± 0.053	0.427 ± 0.033	0.436 ± 0.024	0.471 ± 0.075

Data are represented as means ± SD. Abbreviations: *TA* Tibialis anterior, *EDL* Extensor digitorum longus. *Significant difference compared with male Sham, *P* < 0.05. ^†^*P* = 0.054 compared with male Sham. Data were previously reported [4]

### Muscle fiber size and capillarization

#### Extensor digitorum longus

**Fig. 1:**
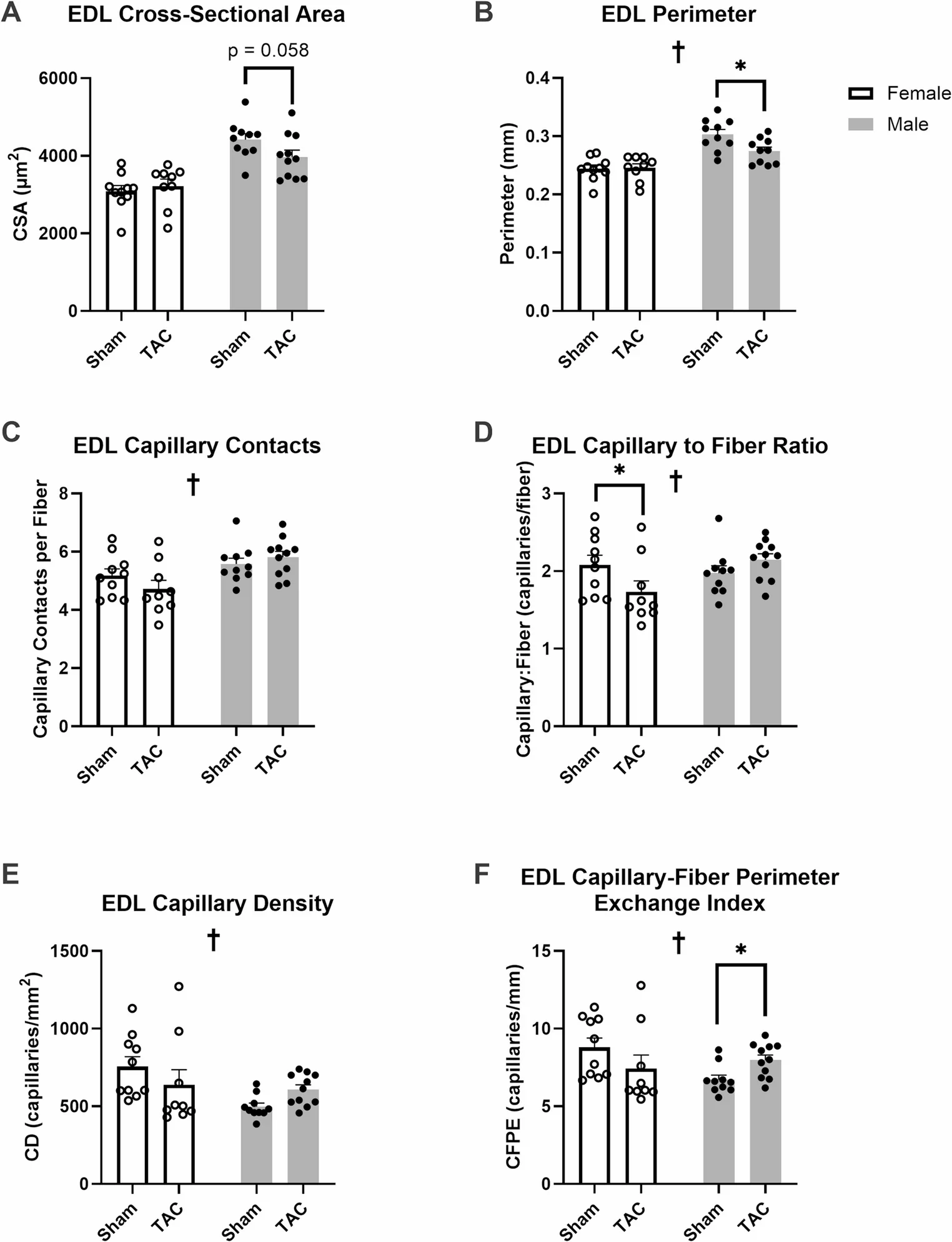
Extensor digitorum longus (EDL) muscles exhibit sex-specific responses in TAC and Sham rats after 40 weeks of progressive pressure overload. Sections of EDL muscle from male (n=21) or female (n=19) TAC (n=20) or Sham (n=20) rats were stained with anti-griffonia simplicifolia Lectin-I and anti-laminin and were imaged with a fluorescent microscope at 200X magnification. Images were analyzed with Muscle2View and verified manually to measure skeletal muscle fiber size (**A**, **B**) and indices of capillarization (**C**-**F**). Data are represented as means and standard errors. †Sex by condition interaction effect (p<0.05 for all but capillary density p=0.058). *p<0.05 for Sham vs TAC within sex. Abbreviations: capillary fiber perimeter exchange (CFPE), capillary density (CD), cross sectional area (CSA). Data in panels A and B were previously reported [4]

As we previously reported [4], the EDL had ~ 10% smaller fiber perimeter and a tendency for smaller EDL fiber cross-sectional area in male TAC vs. Sham rats as shown in Fig. 1A-B. Muscle capillarization was explored as a potential contributor to smaller fiber size (Fig. 1C-F). There were significant sex * condition interaction effects for capillary contacts, capillary to fiber ratio, and fiber perimeter exchange (CFPE) (*p* < 0.05), and a tendency for a sex * condition interaction effect on capillary density (*p* = 0.058) with a general effect of TAC to reduce capillarization in females and increase capillarization in males. When analyzed within sex groups, two of these differences reached significance: There was 17% lower EDL capillary to fiber ratio in female TAC rats compared with female Sham rats (*p* = 0.033), and there was 19% higher capillary to fiber perimeter exchange in TAC male rats compared with Sham male rats (*p* = 0.011). 

#### Soleus

**Fig. 2:**
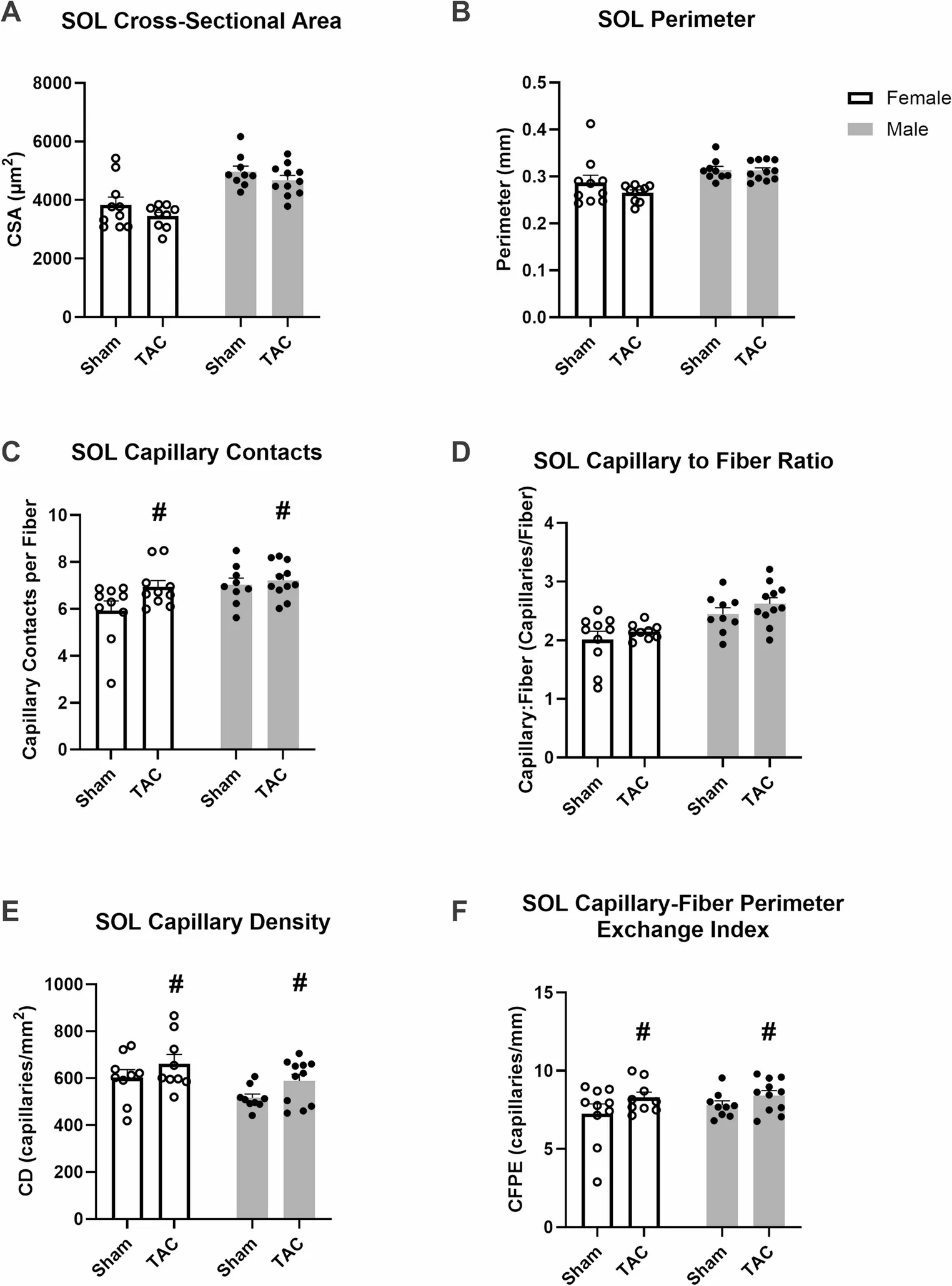
TAC rats have higher soleus (SOL) muscle capillarization after 40 weeks of progressive pressure overload. Sections of soleus from male (*n* = 20) or female (*n* = 19) TAC(*n* = 20) or Sham (*n* = 19) rats were stained with anti-griffonia simplicifolia Lectin-I and anti-laminin and were imaged with a fluorescent microscope at 200X magnification. Images were analyzed with Muscle2View and verified manually to measure skeletal muscle fiber size (**A**-**B**) and indices of capillarization (**C**-**F**). Data are represented as means ± standard errors. There was a main effect of TAC for soleus capillary density (**E**) and CFPE (**F**), and there was a tendency for capillary contacts (**C**) and CSA (**A**) in a two-way ANOVA. ^#^Main effect of TAC (*p* < 0.05). Abbreviations: capillary fiber perimeter exchange (CFPE), capillary density (CD), cross sectional area (CSA). Data in panels **A **and **B **were previously reported [4]

We also measured muscle fiber size and capillarization in the soleus, a muscle with primarily type 1 fibers, to determine whether fiber type differences exist (Fig. 2). As we previously reported [4], soleus fiber CSA and perimeter were not statistically different between TAC or Sham rats, but the numerically lower soleus muscle fiber CSA in TAC vs. Sham rats approached significance (*p* = 0.094). There was a main effect of TAC to have higher capillary contacts, capillary density and CFPE compared with Sham rats. Because CSA, in part, determines capillary density, differences in capillary density could be partially due to the numerically lower soleus muscle fiber CSA. When data were analyzed within sex groups, there were no sex-specific differences detected between TAC and Sham rats for any fiber size or capillarization variable.

### Skeletal muscle immune cell infiltrate

**Fig. 3:**
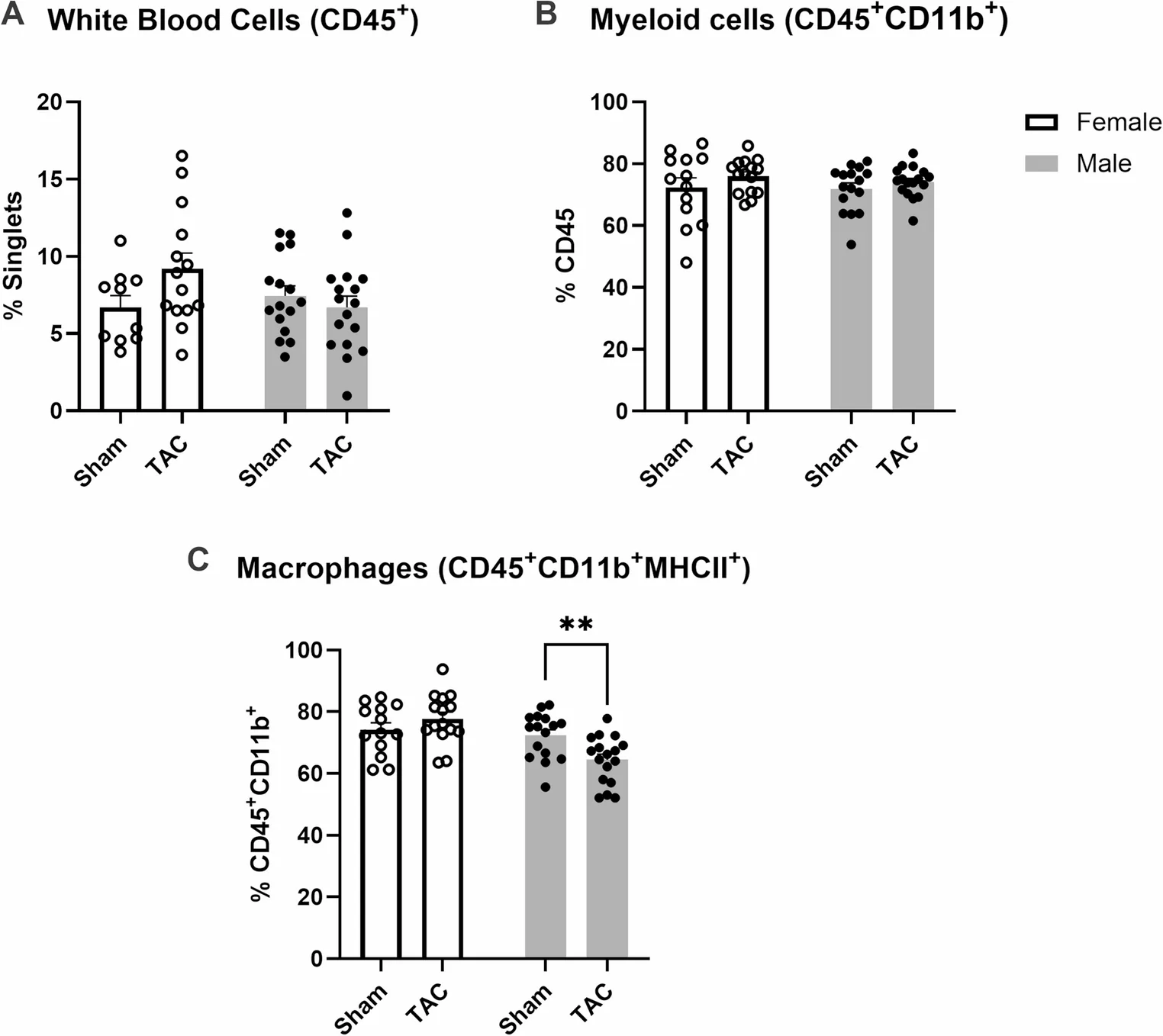
TAC males have lower skeletal muscle macrophage infiltration than Sham counterparts after 40 weeks of transverse aortic constriction (TAC). Rat quadriceps muscles were digested from TAC (*n* = 32) or Sham (*n* = 29) male (*n* = 33) or female (*n* = 28) rats and were stained with antibodies for CD45 **(A)**, CD11b **(B)**, and MHCII **(C)**, and counts were quantified with flow cytometry. Counts are represented as a percent of parent population. Data are represented as mean ± standard error. There was a significant TAC * sex interaction effect for macrophage infiltration (*p* = 0.006). **p = 0.005 for effect of TAC within sex

We measured skeletal muscle macrophage infiltration to determine whether changes in the abundance of immune cells occurred in skeletal muscle as a function of the TAC procedure (Fig. 3). There was a TAC*Sex interaction effect on macrophage infiltration in quadriceps muscle (*p* = 0.006). Sex-specific differences were observed as mean CD45^+^ CD11b^+^ MHCII^+^ macrophage counts were 19% lower in male TAC rats compared with male Sham rats (Fig. 3C, *p* = 0.005). Moreover, TAC had different effects on male and female rats, as the mean percent of CD45^+^ CD11b^+^ MHCII^+^ was ~ 21% higher in female TAC than male TAC. No other differences were noted in skeletal muscle immune cell infiltration.

### Macrophage conditioned medium

#### Tube formation

**Fig. 4:**
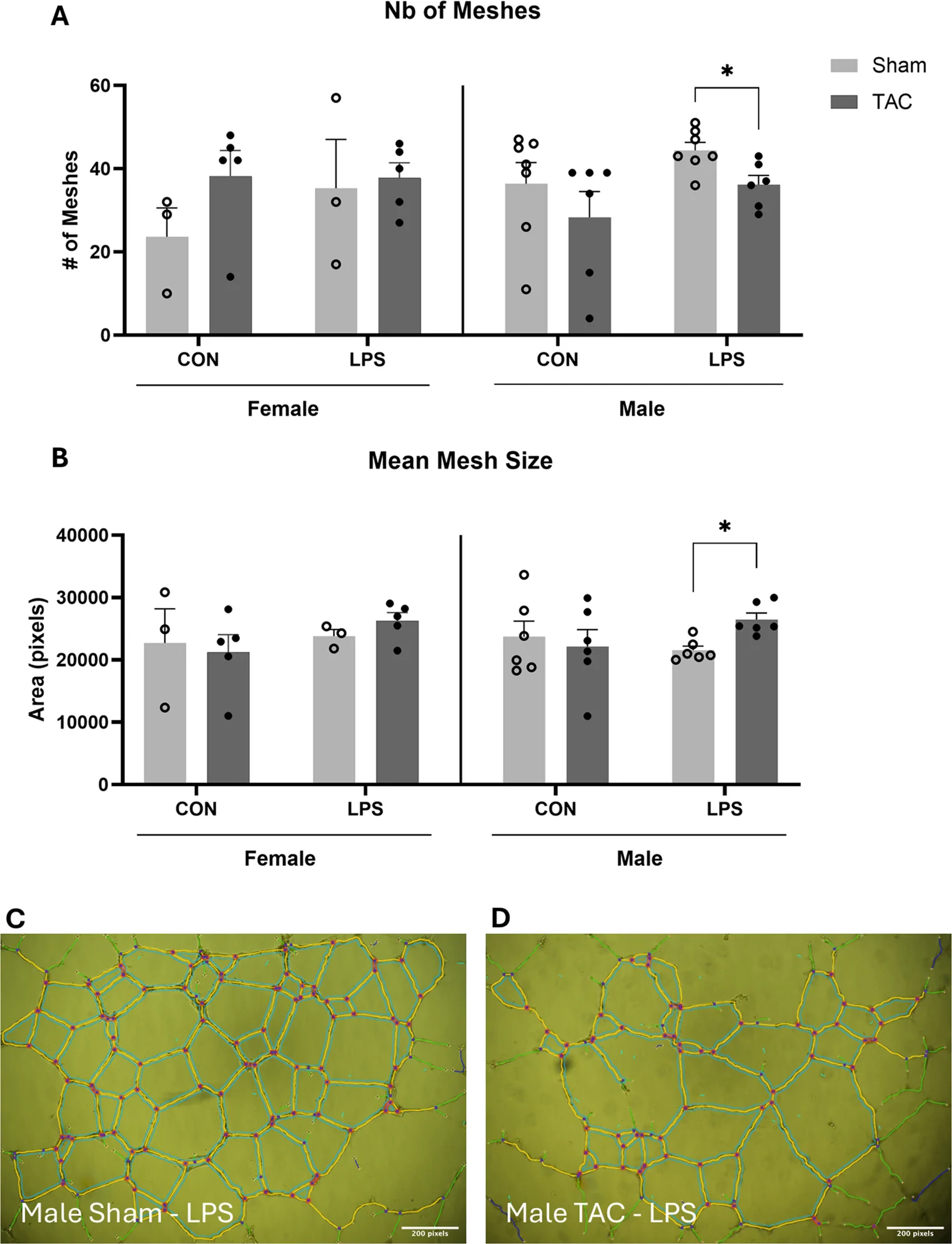
Macrophages from male TAC rats are less pro-angiogenic than male Sham macrophages. Fluorescence-activated cell sorting was performed to purify skeletal muscle macrophages (CD11b+MHCII+) from freshly digested rat quadriceps muscle after 40 weeks of transverse aortic constriction (*n* = 6 male TAC, *n* = 7 male Sham, *n* = 6 female TAC, *n* = 3 female Sham). Purified macrophages were incubated for 48 h with LPS (1 µg/mL) to investigate the inflammatory response compared with an unstimulated control condition (CON). Human umbilical vein endothelial cells were cultured in conditioned medium in wells on top of Matrigel for 24 h, and images were acquired at 40X magnification. Mesh number (**A**) and size (**B**) were quantified with Angiogenesis Analyzer and data are represented as mean and standard error. Representative images from male Sham LPS condition (**C**) and male TAC LPS condition (**D**) are shown. There was a main effect of TAC for mean mesh size in a two-way ANOVA. * *p* < 0.05 for independent effects of TAC or Sham with a treatment and sex. Capillary tubes grown in LPS-stimulated macrophage conditioned medium after 24 h. Abbreviations: Transverse aortic constriction (TAC), lipopolysaccharide (LPS), and control (CON)

To assess the effects of TAC on skeletal muscle macrophage angiogenic potential, we conducted tube formation experiments in which HUVECs were incubated with conditioned medium generated with macrophages isolated from quadriceps muscle (Fig. 4). We also stimulated these macrophages with LPS to determine whether there were differences in the macrophage conditioned medium effects following an inflammatory perturbation. Effects of LPS when TAC and Sham conditions were combined were assessed to illustrate the overall angiogenic effect of LPS stimulation. This showed an overall main effect of LPS to increase total tube segment length compared with control (13,495 ± 424 vs. 12,168 ± 758 pixels, respectively, p = 0.028) and number of meshes (38 ± 2 vs. 33 ± 3 meshes, respectively, p = 0.027). Specific effects of LPS within sex and condition groups are shown in Fig. 4. When stimulated with LPS, macrophages from TAC rats resulted in 18% larger mesh sizes in both sexes combined (26,384 ± 2593 pixels vs. 22,300 ± 1980 pixels, p = 0.001) indicating that TAC macrophages stimulated less dense capillary-like tube networks. These differences appeared to be driven by male rats as male TAC rat LPS-stimulated macrophages elicited 23% fewer meshes (p = 0.022) and 19% larger meshes (p = 0.002) than Sham, respectively. There were no significant differences in capillary-like tube network formation using macrophages from female rats.

#### Macrophage Cytokine Secretion

**Fig. 5:**
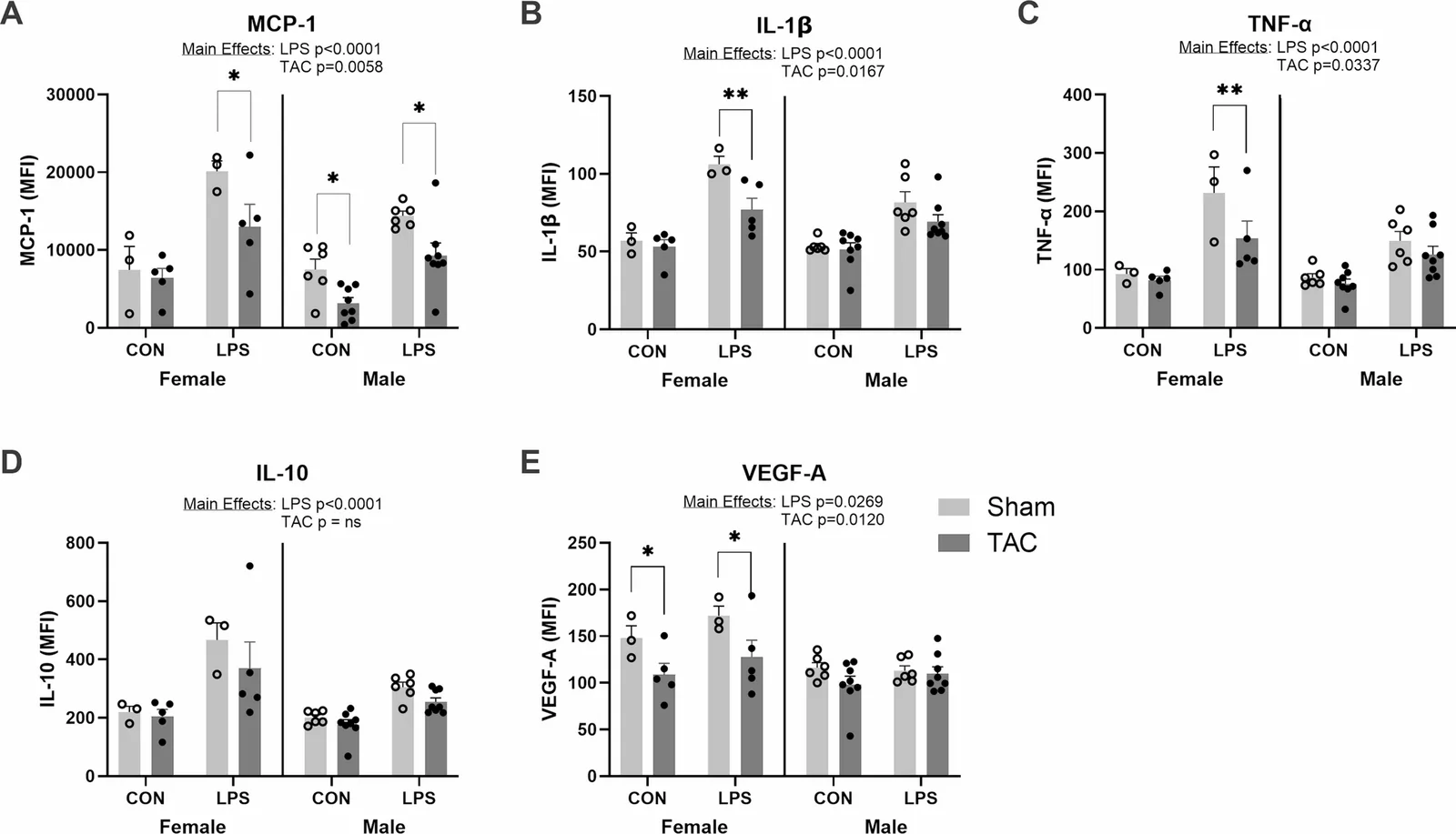
Skeletal muscle macrophages from TAC compared with Sham rats have lower levels of cytokine secretion. Fluorescence-activated cell sorting was performed to purify skeletal muscle macrophages (CD11b+MHCII+) from freshly digested rat quadriceps muscle after 40 weeks of transverse aortic constriction. Purified macrophages were incubated for 48 h with LPS (1 µg/mL) to investigate the inflammatory response compared with an unstimulated control condition (CON). The pro-inflammatory cytokines, MCP-1 (**A**), IL-1b (**B**), and TNFα (**C**), anti-inflammatory cytokine, IL-10 (**D**), and angiogenic factor VEGF-A (**E**), were measured with a multiplex bead assay and mean fluorescence intensity (MFI) is presented. Data are presented as means ± SEM. *p < 0.05, **p < 0.01 for TAC vs. Sham within a sex and treatment. Abbreviations: control (CON), transverse aortic constriction (TAC), interleukin (IL), monocyte chemoattractant protein-1 (MCP-1), Tumor necrosis factor alpha (TNF-α), vascular endothelial growth factor-A (VEGF-A)

After observing significant effects of TAC on the pro-angiogenic potential of macrophages from male rats, we measured the secretion of macrophage paracrine inflammatory factors to characterize the macrophage phenotype (pro-inflammatory or regulatory) and specific proteins potentially responsible for these differences (Fig. 5). Accordingly, we tested for main effects of TAC and LPS administration, then assessed contrasts to compare TAC vs. Sham within each sex and condition. Overall, LPS treatment increased levels of MCP-1 by 132%, IL-1β by 55%, TNF-α by 96%, IL-10 by 74% (p < 0.0001 for all), and VEGF-A by 11% (p = 0.0269) compared with control. In addition, there were main effects of TAC macrophages to decrease MCP-1 by 35% (p = 0.006), IL-1β by 16% (p = 0.017), TNF-α by 17% (p = 0.034) and VEGF-A by 12% (p = 0.012), but not IL-10 compared with Sham macrophages. Male TAC vs. Sham rats and Female TAC vs. Sham rats were then individually compared to determine if the TAC effects differed by sex and more or less evident after an inflammatory stimulus (LPS). In the control condition, only 2 differences were detected: Macrophages from male TAC rats secreted 58% less MCP-1 than male Sham rats (p = 0.0388), and macrophages from female TAC rats secreted 27% less VEGF-A than female Sham rats (p = 0.0288). In the LPS condition, these differences persisted, and additional effects were identified: macrophages from female TAC rats secreted 39% less MCP-1 (p = 0.0130), 28% less IL-1β (p = 0.0026), and 34% less TNF-α (p = 0.093).

## Discussion

Cardiac pressure overload, induced by TAC, was associated with changes in skeletal muscle fiber size and capillarization, as well as macrophage infiltration and phenotype, and some changes occurred in a sex-based manner. In soleus muscles (primarily type 1 fibers), both male and female TAC rats exhibited higher number of capillary contacts, capillary density and CFPE compared with Sham; however, sex differences were observed in EDL muscle (primarily type 2 fibers), with only male TAC rats having smaller muscle fiber size and higher CFPE compared with Sham. Interestingly, TAC male rats had significantly fewer muscle macrophages than all other groups, and this was accompanied by a reduced macrophage secretion of cytokines and lower angiogenic potential after an inflammatory stimulus (LPS). These findings suggest that macrophages may partially mediate skeletal muscle alterations in cardiac pressure overload.

### Skeletal muscle fiber size and capillarization in TAC and Sham rats

Our data support an effect of TAC to increase capillarization in soleus muscle regardless of sex. However, in EDL muscle, we observed a sex-specific effect of TAC to reduce fiber size and increase capillarization only in male rats. Our findings in the soleus are similar in direction and magnitude to previous studies demonstrating lower CSA of soleus fibers in a Dahl salt-sensitive rat model of heart failure vs. control rats [25], though the effect of TAC did not reach significance (*p* = 0.094) in our study. Lower muscle fiber size has previously been shown to correlate with lower capillary to fiber ratios in humans without cardiac dysfunction [20, 21]; however, we observed higher soleus capillary density with slightly smaller fiber sizes in TAC compared with Sham rats. This finding was surprising given that previous studies in humans show lower quadriceps muscle capillary density in those with CVDs like heart failure with preserved ejection fraction [26–28], though mixed results have been reported in humans and rodent models [29, 30].

#### Soleus muscle

In rodent models, previous studies demonstrated mixed results regarding the soleus depending on the model used to mimic heart failure. For example, in Dahl salt-sensitive rats, the soleus exhibits lower muscle fiber size, force production, fatigue resistance, mitochondrial density, and antioxidant capacity [25] along with decreased anabolic and increased catabolic factors [31]. In an obesity-driven model of cardiac dysfunction, the soleus showed muscle atrophy, but had similar fatigability and there was a tendency for greater mitochondrial density [32]. In the present study, TAC rats had greater soleus capillary density due to the combined effect of greater numeric capillary to fiber ratios (numerator) and lower numeric fiber cross sectional areas (denominator), even though these individual variables were not statistically different. Additionally, the modestly lower fiber sizes compared to previous rodent studies are likely because the present TAC rats exhibit less severe cardiac hypertrophy. Collectively, more advanced cardiac dysfunction was present in the previous studies, and this may explain the differences in study findings.

The rats in the present study exhibited very mild cardiac dysfunction [4], making the comparison to clinical populations with heart failure imperfect. In humans with more severe heart failure, reductions in type 1 and type 2 muscle fiber size and decreased capillarization are commonly reported, typically in older populations with mean ages around ~ 70 years [2, 26, 33]. In contrast, our rats were ~ 40 weeks old, whereas aged rats are generally considered 2–3 years old [34], indicating a comparatively younger cohort relative to humans with established heart failure-related capillary rarefaction [2, 26, 33]. Thus, differences in age and disease stage may account for the divergent findings, with preserved or increased capillarization in our model potentially reflecting early compensatory adaptations prior to the more pronounced remodeling seen in advanced human heart failure.

#### Extensor digitorum longus muscle

Few studies have examined muscles primarily comprised of type 2 muscles in pressure overload models. Consistent with the present finding of lower EDL fiber size in TAC vs. Sham rats, studies assessing the EDL or other primarily fast-twitch muscles in monocrotaline-induced heart failure show muscle fiber atrophy [35, 36] along with lower myoD expression, a muscle transcription factor important for maintenance of type 2 fibers [37]. Kaneguchi et al. [36] also reported reduced fiber size and increased collagen content in the fast-twitch muscles from heart failure rats, with no differences in the masses of the muscles when normalized to body weight. While the present study does not include a measure of collagen, our findings of lower EDL fiber size in male TAC rats, with similar muscle masses compared with Sham rats are consistent with that report.

Male TAC rats also had higher CFPE than male Sham rats. Because the number of capillary contacts was similar between male Sham and male TAC while the fiber size was smaller in male TAC rats, the greater EDL capillarization in male TAC rats appears driven by lower fiber size rather than an actual increase in the number of capillaries. Female TAC rats, in contrast, did not exhibit this change, with similar muscle fiber size and lower capillary-to-fiber ratio, suggesting sex-specific differences in muscle remodeling and potential capillary rarefaction.

### Skeletal muscle macrophages

This is one of the first studies to show differences in skeletal muscle macrophage infiltration and phenotype induced by cardiac pressure overload. We report that TAC males have the lowest number of skeletal muscle macrophages while female TAC rats show no differences compared with Sham counterparts. To our knowledge, only one other study has evaluated skeletal muscle macrophages in humans with cardiac dysfunction, and showed no significant differences in macrophage infiltration, though the methods of measurement may explain these differences [38]. Specifically, the aforementioned study performed immunohistochemistry to count macrophages within the muscle, which may not detect differences across a skeletal muscle. Conversely, we employed whole tissue digestion and flow cytometry that would encompass potentially heterogenous regions of muscle inflammation.

The macrophages from TAC compared with Sham rats exhibited immunosuppressive-like characteristics. TAC rats secreted lower levels of proinflammatory, anti-inflammatory, and growth promoting cytokines, which are more in line with the effects of aging [39, 40]. There are few studies investigating changes in skeletal muscle macrophages in CVDs such as heart failure; however, there are comparative data in cardiac tissue of those with severe heart failure where accumulations of pro-inflammatory macrophages in the heart correlate with reduced ejection fraction and progression to ventricular dilation [41]. While these findings conflict with ours in terms of inflammatory direction, they do show that chronic diseases can affect macrophage infiltration, which supports the notion that macrophage infiltration is influenced by disease state and is a plausible pathway explaining peripheral dysfunction [42].

#### Tube formation

In tube formation experiments, conditioned medium from macrophages of TAC males produced less complex capillary networks with fewer and larger capillary meshes than Sham males. In females, there were no differences in tube formation between groups. In males, the lower LPS-stimulated tube formation in TAC compared with Sham does not align with the greater capillarization of muscle from male TAC rats when compared to male Sham rats. The apparent incongruence between capillary density and tube formation may be due to what is actually measured in the capillary-like tube formation assay (ex vivo capillary formation and organization) versus histological assessment of skeletal muscle. It is important to note that because skeletal muscle fibers were atrophied in TAC, this inflated some capillarization metrics in TAC rats but does not represent an increase in in vivo angiogenesis.compared with Sham rats. Capillary organization can influence oxygen consumption by altering RBC velocity, flux and the percent of capillaries supporting continuous flow [43]. Thus, the less complex capillary networks produced by TAC macrophages may indicate organizational changes in capillary networks that eventually result in lower capillary densities.

#### Cytokines

Skeletal muscle macrophages from TAC rats secreted lower levels of all cytokines compared with sham rats, and this change was exaggerated after stimulation with LPS. This was surprising given studies demonstrating elevated systemic inflammation in cardiovascular diseases and a Dahl salt-sensitive rat model of heart failure with preserved ejection fraction [44]. However, the rats in the present study were relatively older and exhibited a less severe cardiac phenotype compared with these models. In aging and diseases such as type 2 diabetes, muscle macrophages adopt similar regulatory phenotypes and secrete less IL-1β and IL-10 after muscle damage via exercise [39, 40, 45, 46]. While skeletal muscle was not intentionally damaged to elicit an inflammatory response in this study, LPS was used to measure the macrophage inflammatory response, and the production of cytokines in our TAC rats was attenuated compared with Sham rats. These results suggest macrophages may mimic changes with aging and diabetes – common risk factors for CVDs.

The reduction in macrophage secretion of inflammatory cytokines in TAC rats compared with Sham rats could contribute to muscle atrophy. Specifically, MCP-1 is a potent chemokine secreted by macrophages immediately following signaling through damage or pathogen associated molecular pattern to initiate a robust early inflammatory response [9, 47]. In the absence of MCP-1 receptors (CCR2) on skeletal muscle, there is subsequent muscle fiber atrophy and impaired angiogenesis [9]. Lower MCP-1 may likely preferentially effect type 2 muscle fibers, as the EDL muscle is susceptible to delirious effects of MCP-1 knockout than soleus muscle [48]. This is also concordant with the aforementioned study by Kaneguchi et al. [36] wherein monocrotaline-induced heart failure primarily impacted type 2 skeletal muscle fibers.

With respect to anti-inflammatory and growth promoting cytokines, macrophages from male rats secreted relatively low amounts of VEGF-A and IL-10. Even after stimulating these macrophages with LPS to increase IL-10 and VEGF-A, the secretion from male rat macrophages was similar to female TAC macrophages (that also exhibited some blunting) without any stimulation. This finding represents an overall repression of cytokine secretion in TAC rats irrespective of whether the cytokine is proinflammatory, anti-inflammatory or growth promoting, particularly in Male TAC rats. Collectively, macrophages from TAC rats appear less active.

### Sex differences in TAC and Sham rats

Male TAC rats had smaller EDL (primarily type 2) muscle fibers and higher capillary density compared with Sham males, whereas female TAC rats showed no change in EDL fiber size or capillarization. In soleus (primarily type 1) muscles, both sexes demonstrated increased capillarization relative to Sham controls, indicating a shared adaptation in oxidative fibers. However, males and females diverged markedly in their immune responses: TAC males had significantly fewer skeletal muscle macrophages and displayed a blunted cytokine and chemokine secretion profile, while TAC females maintained macrophage numbers and produced higher levels of inflammatory and growth-promoting cytokines, including VEGF-A. The absence of macrophages contributes to poor muscle regeneration, cell death, skeletal muscle fiber atrophy, immune infiltrate and tissue fibrosis [47]. Functionally, macrophages from male TAC rats exhibited lower angiogenic potential and generated less complex capillary networks in vitro compared with those from females or Sham males. Collectively, these results suggest that male rats develop a more immunosuppressed and atrophy-prone skeletal muscle phenotype under pressure overload, whereas females retain a more active immune and angiogenic response that may confer relative protection against muscle remodeling.

Finally, macrophage cytokine secretion appeared to be uncoupled from endothelial tube formation. VEGF-A, a key driver of angiogenesis, did not differ significantly between TAC and Sham males under either basal or LPS-stimulated conditions. In contrast, female TAC rats had significantly lower VEGF-A levels than their Sham counterparts. Based on these findings, one would expect reduced tube formation in female TAC macrophage media, with little or no difference in males. However, the opposite pattern emerged: only male TAC macrophages produced less complex capillary networks following LPS stimulation, while females showed no difference between groups. One possible explanation is that overall VEGF-A secretion in males was lower than in females across conditions, potentially placing male VEGF-A levels near a physiological “threshold” below which angiogenic processes are more sensitive to change. In contrast, VEGF-A concentrations in females may have remained above this threshold, resulting in preserved tube formation despite lower VEGF-A secretion. This threshold-dependent response could explain why angiogenic impairment was observed in males but not females.

### Strengths and limitations

This is one of the first studies to use whole skeletal muscle tissue to comprehensively assess both macrophage infiltration, cytokine secretion, and paracrine angiogenic function in a TAC model that accounts for muscles predominately comprised of either type 1 or type 2 fibers in both male and female rats. The study does, however, have some limitations. First, our study isolated macrophages from the quadriceps, and we analyzed capillarization and fiber sizes in the soleus and EDL. Isolation of macrophages and histological assessments from the same muscle were not feasible because a large muscle sample was required to obtain enough muscle macrophages for functional analyses, and this limited the sample available for histology. Furthermore, we did not perform an in-vivo perturbation to inflame the skeletal muscle, which may not represent the physiological state of macrophages in those with heart failure. Second, the tube formation assay used as an index of angiogenesis has limitations and does not capture all endothelial cell functions necessary for in vivo angiogenesis. Regardless, differences among conditions and groups still represent valid experimental differences as a function of macrophage conditioned medium. Third, we also cannot rule out the possibility of different relative degrees of TAC in male vs. female rats in this study as our previous report [4] showed that the 40-week-old male Sham rates had ~ 0.4 mm larger aortic arch diameters than 40-week-old female Sham rates. However, there is conflicting evidence on the impact of the degree of constriction on heart failure phenotypes in TAC models [49, 50], and another report from our lab using this model indicates that TAC imposed increased vascular resistance in both sexes [51]. Finally, while we cannot completely rule out a differential contribution of aging across sexes and conditions, the inclusion of Sham animals followed in the same time course help to eliminate this as a possibility.

## Conclusions

A moderate degree of TAC is associated with sex-specific skeletal muscle adaptations in rats. Male TAC rats exhibit reduced EDL (type 2) fiber size with preserved capillarization compared with male Sham rats, compensatory increases in soleus (type 1) fiber capillarization, and reduced macrophage infiltration of quadriceps muscle compared with female TAC rats. In male TAC rats, skeletal muscle macrophages also display a less angiogenic phenotype following LPS stimulation, producing less developed capillary networks ex vivo. These findings suggest that cardiac pressure overload-induced early immune and microvascular remodeling may differ by sex and these outcomes should be further investigated to establish potential targets for therapeutic intervention.

## Supplementary Information

Below is the link to the electronic supplementary material.

Supplementary Material 1

## Data Availability

Data are available from the authors upon reasonable request.
