# Supplementary material for: Sex-specific skeletal muscle fiber atrophy and reduced macrophage inflammation in a rodent transverse aortic constriction model
Source: Pflugers Arch. 2026 Oct 10;478(11):87. doi: 10.1007/s00424-026-03220-w (PMC13653819; doi:10.1007/s00424-026-03220-w)
Supplement: Supplementary file 1 [file 424_2026_3220_MOESM1_ESM.docx]

**Sex-specific skeletal muscle fiber atrophy and reduced macrophage inflammation in a rodent transverse aortic constriction model**

William S. Evans, Maria Canellas da Silva, Gabriel Pena, Sarah Kuzmiak-Glancy, and Steven J. Prior

**Supplement**

**Supplemental Figure 1. Representative flow-cytometry gating strategy.** Numbered arrows show the parent-to-child gating hierarchy: (1) total events displayed by forward scatter area (FSC-A) and side scatter area (SSC-A), (2) singlets selected using forward scatter height (FSC-H) versus FSC-A, (3) CD11b-positive events identified from Pacific Blue fluorescence, and (4) MHCII-positive events identified within the CD11b-positive population using PerCP fluorescence, followed by the final Pacific Blue-versus-PerCP display of the MHCII-positive population. Horizontal gate markers indicate the thresholds used to distinguish negative and positive populations; percentages denote the proportion of events within the corresponding parent population.

**A) Soleus	B) Extensor Digitorum Longus**

**Supplemental Figure 2. Example images of immunostained soleus (A) and extensor digitorum longus (B) muscles.** Capillaries were detected with *Griffonia Simplicifolia Lectin I* Dylight 594 (Vector Laboratories Cat# DL-1207, RRID:AB_2336415) (red/orange) and fiber perimeters were detected with rabbit anti-rat Laminin (Abcam Cat# AB_11575, RRID:AB_298179) (green). Images were acquired at 200X and these sample images represent a portion of the cross-section of muscle analyzed.
